# Effects of Mountain Ultra-Marathon Running on ROS Production and Oxidative Damage by Micro-Invasive Analytic Techniques

**DOI:** 10.1371/journal.pone.0141780

**Published:** 2015-11-05

**Authors:** Simona Mrakic-Sposta, Maristella Gussoni, Sarah Moretti, Lorenza Pratali, Guido Giardini, Philippe Tacchini, Cinzia Dellanoce, Alessandro Tonacci, Francesca Mastorci, Andrea Borghini, Michela Montorsi, Alessandra Vezzoli

**Affiliations:** 1 Institute of Bioimaging and Molecular Physiology, National Council of Research (CNR), Segrate (Milan), Italy; 2 Department of Pathophysiology and Transplantation−Physiology Section, University of Milan, Milan, Italy; 3 Institute of Clinical Physiology, National Council of Research (CNR), Pisa, Italy; 4 Neurology and Neurophysiology Department. Mountain Medicine Center Valle d’Aosta Regional Hospital Umberto Parini, Aosta, Italy; 5 EDEL Therapeutics S.A., PSE-B/EPFL, CH-1015 Lausanne, Switzerland; 6 Università Telematica S. Raffaele Roma, Italy; University of the Balearic Islands, SPAIN

## Abstract

**Purpose:**

Aiming to gain a detailed insight into the physiological mechanisms involved under extreme conditions, a group of experienced ultra-marathon runners, performing the mountain Tor des Géants® ultra-marathon: 330 km trail-run in Valle d’Aosta, 24000 m of positive and negative elevation changes, was monitored. ROS production rate, antioxidant capacity, oxidative damage and inflammation markers were assessed, adopting micro-invasive analytic techniques.

**Methods:**

Forty-six male athletes (45.04±8.75 yr, 72.6±8.4 kg, 1.76±0.05 m) were tested. Capillary blood and urine were collected before (Pre-), in the middle (Middle-) and immediately after (Post-) Race. Samples were analyzed for: Reactive Oxygen Species (ROS) production by Electron Paramagnetic Resonance; Antioxidant Capacity by Electrochemistry; oxidative damage (8-hydroxy-2-deoxy Guanosine: 8-OH-dG; 8-isoprostane: 8-isoPGF2α) and nitric oxide metabolites by enzymatic assays; inflammatory biomarkers (plasma and urine interleukin-6: IL-6-P and IL-6-U) by enzyme-linked immunosorbent assays (ELISA); Creatinine and Neopterin by HPLC, hematologic (lactate, glucose and hematocrit) and urine parameters by standard analyses.

**Results:**

Twenty-five athletes finished the race, while twenty-one dropped out of it. A significant increase (Post-Race vs Pre) of the ROS production rate (2.20±0.27 vs 1.65±0.22 μmol^.^min^-1^), oxidative damage biomarkers (8-OH-dG: 6.32±2.38 vs 4.16±1.25 ng^.^mg^-1^ Creatinine and 8-isoPGF2α: 1404.0±518.30 vs 822.51±448.91 pg^.^mg^-1^Creatinine), inflammatory state (IL-6-P: 66.42±36.92 vs 1.29±0.54 pg^.^mL^-1^ and IL-6-U: 1.33±0.56 vs 0.71±0.17 pg^.^mL^1^) and lactate production (+190%), associated with a decrease of both antioxidant capacity (-7%) and renal function (i.e. Creatinine level +76%) was found.

**Conclusions:**

The used micro-invasive analytic methods allowed us to perform most of them before, during and immediately after the race directly in the field, by passing the need of storing and transporting samples for further analysis. Considered altogether the investigated variables showed up that exhaustive and prolonged exercise not only promotes the generation of ROS but also induces oxidative stress, transient renal impairment and inflammation.

## Introduction

Under extreme conditions, including those exerted by extreme environments and/or exercise, human body is pushed to its limits. Competitions such as ultra-running race are close to the limits of human performance so that exercise physiologists are very interested in analyzing the mechanisms of sports where runners compete over very long distances. Acute physiological stress, orthopedic trauma accompanied by muscle injuries, cardiac risk, oxidative stress damage, systemic inflammation, immunologic alterations, inflammatory responses and DNA damage may occur in intensive exercise such as ultra-endurance, ultra-distance, marathon, ultra-marathon race events [1,2]. For all these reasons, ultra-sports can provide useful ground for medical and biological investigations [3]. Over the last 10 years, there has been an exponential increase in the number of published, peer-reviewed studies, and conferences about ultra-running worldwide. Popular in Europe, ultra-marathon is any sporting event involving running and walking longer than the traditional marathon length (26.2 miles in a time ranging between 2 and 6 hours).

As well established, oxidative stress research indicates that ultra-endurance exercise results in the formation of Reactive Oxygen Species (ROS) [4]. As a consequence, an increase in oxidative stress (OxS) takes place, able to potentially overwhelm antioxidant defenses [5–7]. ROS term is referred to radicals and non-radicals compounds, derived from oxygen, that have the ability to quickly react with cell components, damaging lipids, proteins, carbohydrates, nucleic acids [8,9] and perturbing cellular mechanisms. Nonetheless, at appropriate concentration, ROS are known to act as important signaling molecules [10,11].

Numbers of sources of ROS have been identified during exercise, including the mitochondrial electron transport chain, hemoglobin and myoglobin, NADPH oxidase and catecholamine autoxidation [8,12,13]. Many approaches allow demonstrating ROS participation in biochemical events and different methods are reported for their quantification. The measurement of ROS is difficult because they are produced in low quantities, are endowed with a very short half-life and a rapid interaction with antioxidants, and cellular components like thiol residues, molecular oxygen, and metalloproteins. Indirect enzymatic methods have been developed for measuring ROS-induced damage, based on the determination of specific end products resulting from the interaction of ROS with biological macromolecular targets [8,9,14]. However enzymatic assays can be considered ‘a posteriori’ methods with respect to Electron Paramagnetic Resonance (EPR) technique, able to provide direct detection of the ‘instantaneous’ presence of free radical species in the sample [10]. EPR results the method of choice, since it is the only technique capable of returning, in a micro-invasive way, a ‘intrinsic’ quantitative information. In fact, in EPR spectra, signal areas are proportional to the number of the excited electron spins, leading to absolute concentration levels, when adopting a stable radical compound as reference. Nevertheless ROS half life is too short (superoxide [O_2_^·-^] t_1/2_ (s): 10^−4^; Nitric oxide [NO·]: 0.4 s at ambient temperature), if compared to the EPR time scale, so they are EPR-invisible: only when ‘trapped’ and transformed in a more stable radical species they become EPR detectable [15–18].

Several studies have documented that exercise-induced muscle damage is accompanied by the presence of inflammatory mediators and this modulation appears to be affected by intensity, mode and duration of the exercise challenge [19,20]. In particular, ROS production by immune cells is dependent upon a numbers of hormones and cytokines. For example interleukin-6 (IL-6) is produced in large amount in contracting skeletal muscles and released into the circulation [21,22].

Another marker providing information about immune activation, controlled by T helper cells type1, is urinary-Neopterin [23]. Neopterin is predominantly produced by human monocytes/macrophages, following a functional block of the tetrahydrobiopterin synthesizing pathway, so that it has been used as an inflammation surrogate marker [23].

Altered renal functions have also been often reported in various sports, including prolonged and strenuous exercise, such as marathon running [24]. Such impairment resulted in a significant increase of Creatinine, Urea, and Uric Acid [24,25]. Proteinuria may be a sign of kidney disorders, but its temporary increase in marathon running is considered to be normal. Reid RD et al. [26] reported that proteinuria occurred in the 35% and 69%, while hematuria, with or without proteinuria, in the 21% and 22% of the athletes running the half and the full marathon, respectively [26]. Likewise, urinary excretion of Nitric Oxide (NO) metabolites (nitrates + nitrites) also increases when raising the level of physical activity [27].

Aiming to gain a better insight into the physiological mechanisms involved under extreme conditions, when the human body is pushed to its limits, a group of athletes performing the mountain ultra-marathon (Tor des Géants®) was monitored. In particular, as is well known, Redox Physiology plays an important role in the control of performance, especially in endurance exercise, as ultra-marathon race. Cell redox status modifications under this exercising condition were investigated in the present study, by following selected oxidative stress/redox markers before, during and immediately after the ultra-marathon race so that its impact on the redox physiology/balance could be documented. Indeed modifications of the cell redox balance have been already reported in the literature [5,7]. Nonetheless, and this was a further purpose of the study, only micro-invasive methods were adopted (just capillary blood by fingertip and urine were collected for the measurements). This choice becomes of paramount importance when studies are carried out on athletes and/or in extreme environments. Moreover EPR technique was for the first time adopted in ultra-marathon runners. The study was based on the evaluation of several OxS markers: ROS production rate, antioxidant capacity, oxidative damage biomarkers, inflammatory response and functional renal impairment, coupled with anaerobic response analysis throughout the measurement of lactate production. Possible correlations between ROS production levels and the other parameters were also investigated.

## Materials and Methods

### Snapshot of the race

**Fig 1:**
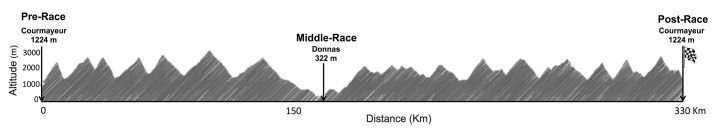
Elevation profile of the race (TDG). (http://www.tordesgeants.it/it/content/percorso).

The study was performed during two consecutive editions of the extreme mountain ultra-marathon (MUM) of the world “Tor des Géants” (TDG), organized in September 7–14^th^ 2013 and 2014; 330 km, an altitude difference of 24,000 m (3 times the Everest), 25 mountain passes above 2000 m s.l., running in just one single stage in the heart of the "Giants" of Valle d'Aosta (Italy). Run along the route of the two Alte Vie, the Tor des Géants is a journey amid the highest peaks in Europe, winding its way along at the foot of Mont Blanc (4810 m), the Gran Paradiso (4061 m), Monte Rosa (4634 m) and the Cervino (4478 m): the four Giants of the Alps (http://www.tordesgeants.it/it/content/percorso). The 2013 edition had 706 starters and 383 finishers (54%); the 2014 edition 738 starters and 444 finishers (60%). The maximum time allowed for race completion was 150h: the current record of the “King of Gèants” was 70h 04 min 15 sec (2013). In 2014 the record time was 71h 49 min 10 sec. The distance is divided into seven stages with thirty-five aid-relax stations where runners can rest and/or sleep. The organizing committee does not impose rules regarding rest stops, and the winner is the runner who completes the race in the shortest time, deciding by himself when and how long to stop for resting and feeding. Fig 1 shows the elevation profile of the race.

### Subjects

Forty-six experienced ultra-marathon runners (males, age 45.04±8.75 yr, height 1.76±0.05 m) voluntarily participated in the study. Twenty-six subjects were enrolled during the 2013 edition, the other twelve in the 2014 one. The enrollment in 2014 ed. was forced in order to ensure a sample size able to potentially overcome the results attained in the 2013 edition.

Registration to TDG does not met specific limitations, even if the organizing committee strongly recommends to have participated in other long trails taking place over several days, before participating in this trial. During the pre-race session, a questionnaire was administered to collect data on the subjects’ training experience. On average, the subjects had 6.9±3.5 years of ultra-endurance experience. The training sessions in preparation to the race consisted of 4–5 sessions per week (about 68±2.5 km/wk). No specific limitations were imposed to the use of vitamin/minerals supplements, herbs and medications.

A complete assessment of healthy status (e.g. hypertension, hypercholesterolemia, diabetes, smoking habits) of each participant was performed. All athletes were no smokers and in good health. The anthropometric parameters, body mass index (BMI), body fat, free masses were assessed. The total body water (TBW) was determined by bipolar bio-impedentiometry (TBF-300A Body Composition Analyzer; Tanita Corporation, Arlington Heights, IL, USA). Blood pressure (BP) was measured by a standard cuff sphygmomanometer, finger O_2_ saturation (SaO_2_) and Heart Rate (HR) (HR and SaO_2_: Oximetry—Ohmeda TuffSat—GE Healthcare, Helsinki, Finland) were measured too (see Table 1). Measurements were performed all together at rest and immediately after stopping at the Middle and Post Race times. All subjects were fully informed of the procedure and the risks involved and, before data collection, signed a written informed consent outlining study requirements. The procedure was conducted according to the Declaration of Helsinki and approval was obtained from the institutional Ethics Committee of the Aosta Hospital, Italy.

**Table 1:** Anthropometric and physiological parameters of all athletes.

	ALL ATHLETS (n = 46)	FR (n = 25)			NFR (n = 21)	
	Pre-Race	Pre-Race	Middle-Race	Post-Race	Pre-Race	Middle-Race (n = 8)
**Weight (Kg)**	72.6±8.4	72.4±7.9	71.0±7.9¶	70.5±7.8¶	72.8±9.1	72.6±7.3
**BMI (kg** ^.^ **m** ^**-2**^ **)**	23.3±2.6	23.3±2.2	22.9±2.1	22.6±2.3¶	23.3±3.0	23.1±2.8
**Fat Mass (kg)**	5.7±2.7	5.2±2.2	3.6±1.7¶	2.8±1.9¶	6.3±3.1	4.8±2.8
**Fat Free Mass (kg)**	67.2±5.9	67.3±6.3	67.7±6.2	69.2±7.3§	66.5±5.5	67.8±5.6
**TBW (kg)**	49.02±4.9	49.2±4.7	49.1±4.6	50.4±5.3	51.4±3.3	50.3±3.5
**SaO**_**2**_ **(%)**	96.6±1.1	96.4±1.1	97.2±0.8#	96.3±2.2	96.8±1.1	97.1±1.4
**HR (BPM)**	67.3±12.2	70.1±11.8	83.0±10.0§	83.6±10.4¶	64.1±12.1	82.8±11.3§
**Systolic Blood Pressure (mmHg)**	129.3±13.6	128.8±14.7	135.7±11.4	144.1±15.5¶	129.8±12.5	138.5±17.1
**Diastolic Blood Pressure (mmHg)**	77.5±8.2	76.2±8.4	81.9±4.6§	86.4±7.1§	79.1±7.9	87.8±17.3§

Mean (±SD) of the parameters collected from the athletes taken altogether at Pre-Race or referred to the FR and NFR groups at different racing times. BMI: Body Mass Index; TBW: Total Body Water. SaO_2_: arterial Oxygen Saturation. HR: Heart Rate.

Significant differences compared to Pre-Run: P<0.01 (# symbol), P<0.001 (§ symbol), P<0.0001 (¶ symbol).

### Blood and Urine samples collection

Subjects underwent three test sessions for capillary blood and urine samples collection: the first (Pre-Race) was performed 1–2 days preceding the race in Courmayeur, the second (Middle-Race, 148.7 km) at an almost intermediate point in Donnas, the last (Post-Race) immediately followed the end of the race in Courmayeur. In every session (Pre, Middle and Post Race), for each recruited subject, capillary blood (480 μL): was taken from the fingertip: ROS production rate, antioxidant capacity, lactate, glucose concentrations and hematocrit were determined. Plasma samples were obtained by centrifugation of heparinized capillary blood: plasma was aspirated by using micro-loader tips 0,5–20 μL (Eppendorf, U.S.A.) The samples were collected in triplicate. Urine samples were collected Pre and Post race by voluntary voiding in a sterile container provided to the subjects. All samples were stored in multiple aliquots at -80°C until assayed. Samples were thawed only once before analysis, performed within two weeks from collection.

### Blood measurements

#### Oxidative stress (OxS)

ROS detection. A X-band EPR instrument (E-Scan-Bruker BioSpin, GmbH, MA USA) was adopted for ROS determination. The instrument allows us to deal with very low amounts of paramagnetic species in small (50 μL) samples. Among spin probe molecules able to trap the ROS, CMH (1-hydroxy-3-methoxycarbonyl-2,2,5,5-tetramethylpyrrolidine) probe was adopted as the most suitable for biological utilization. ROS production rate was determined by means of a recently developed EPR method [17,18] using blood samples (50μL) immediately treated with CMH solution (1:1). 50μL of the obtained solution was put in a glass EPR capillary tube (Noxygen Science Transfer & Diagnostics, Germany) placed inside the cavity of the E-Scan spectrometer for data acquisition. Acquisition parameters were: microwave frequency: 9.652 GHz; modulation frequency: 86 kHz; modulation amplitude: 2.28 G; sweep width: 60 G, microwave power: 21.90 mW, number of scans: 10; receiver gain: 3.17^.^10^1^. Sample temperature was firstly stabilized, and then kept at 37°C by the Temperature & Gas Controller "Bio III" unit, interfaced to the spectrometer. EPR measurements allowed us to attain a relative quantitative determination of ROS production rate in samples. All data were, in turn, converted in absolute concentration levels (μmol · min^-1^) by adopting CP^•^ (3-Carboxy-2,2,5,5-tetramethyl-1-pyrrolidinyloxy) stable radical as external reference. All spectra were collected by adopting the same acquisition parameters and handled by using the software standardly supplied by Bruker (Win EPR System, V. 2.11).

Antioxidant capacity. Blood Reducing Capacity was measured on a capillary blood sample (10μL), using a commercial EDEL potentiostat electrochemical analyser (Edel Therapeutics, Switzerland) equipped with a redox sensor in a three-electrode arrangement. The technique is an electrochemical-based method responding to all water-soluble compounds in biological fluids, which can be oxidized within a defined potential range [28,29] A screen-printed working carbon electrode (WE), operating in conjunction with a screen-printed counter and a silver/silver-chloride (Ag/AgCl) reference one was used. The blood sample was loaded onto a chip and a potential, increasing from 0 to 1.2 V, at a scan rate of 100 mV^.^s^-1^ (versus Ag/AgCl reference electrode), was applied, while the resulting current was measured at the WE. The result was then pseudo-titrated to account for the most biologically relevant antioxidants [30]. Data are expressed in nW.

#### Enzymatic assays

8-hydroxy-2-deoxy Guanosine (8-OH-dG). 8 hydroxy-2-deoxy guanosine (8-OH-dG) has been established as a marker of oxidative DNA damage. A commercially available enzyme immunoassay kit (EIA, Cayman Chemical, U.S.) was utilized. The kit employs an anti-mouse IgG-coated plate and a tracer consisting of an 8-OH-dG-enzyme conjugate. This antibody-8-OH-dG complex binds to goat polyclonal anti-mouse IgG that has been previously attached to the well. The plate is washed to remove any unbound reagents, and then Ellman's Reagent (which contains the substrate to AChE) is added to the well and the product of this enzymatic reaction absorbs at 412 nm. The sample 8-OH-dG concentration is determined using a 8-OH-dG standard curve.

8-isoprostane (8-iso PGF2α). 8-isoprostane (8-iso PGF2α) concentrations were measured using a commercially available enzyme immunoassay kit (Cayman Chemical, U.S.). Briefly, 50μL of urine samples were placed in 96-well plate that was pre-coated with mouse monoclonal antibody. Thereafter, 50μL of 8-iso PGF2α-tracer and 8-iso PGF2α-antiserum were added into each well and incubated for 18 h at 4°C. After washing with buffer, 200μL of Ellman's reagent containing the substrate of acetylcholinesterase was added. The plates were read at a wavelength between 405 and 420 nm. The lower detection limit of the assay was 0.8 pg^.^mL^-1^. The sample 8-iso PGF2α concentrations were determined using 8-iso PGF2α standard curve.

#### Metabolic and inflammatory markers

Lactate. Capillary blood (20 μL) was obtained from a finger tip for the determination of blood lactate concentration ([La]_b_) by enzymatic method (Biosen 5030; EKF, Eppendorf Italia, Milano, Italy).

Glycaemic test. Blood glucose levels were tested by an electronic device Glucose Accu-Chek® Active test strip (Roche diagnostic) on a drop of blood.

Hematocrit (Hct). Percentage change in plasma volume was determined in capillary blood samples by centrifuging heparinized blood in micro-hematocrit capillaries (Hirschmann Laborgeräte GmbH & Co; Germany) at 10.000 rpm for five minutes (4223 Centrifuge ALC, Sacem S.r.l.; Italy) and directly analyzed for hematocrit. Blood samples were analyzed in duplicate.

Interleukin 6-Plasma (IL-6-P). IL-6-P levels were determined by ultrasensitive ELISA kit (R&D Systems, Minneapolis, MN, USA), according to the manufacturer’s instruction. Briefly, a monoclonal antibody specific for IL-6 was pre-coated onto a microplate. Standards and samples (~200μL) were pipetted into the wells and the immobilized antibody bound any IL-6 present. Following the washing procedure, an enzyme-linked, specific for IL-6, polyclonal antibody was added to the wells. After subsequent washing, a substrate solution was added to the wells and colour developed in proportion to the amount of IL-6 bound at the initial step. The signal was then spectrophotometrically measured at a wavelength of 450 nm.

### Urine measurements

Any urinary marker is known to change considerably over time so that, when the collection of the 24h urine is not possible, urinary parameters are standardized basing on the amount of the excreted creatinine. In fact, in a human subject and in the absence of renal disease, the excretion rate of creatinine keeps relatively constant.

Creatinine and Neopterin. Creatinine and Neopterin concentrations were measured by an isocratic high-pressure liquid chromatography (HPLC) method. Briefly, urine samples were thawed and centrifuged at 13000 rpm for 5 min at 4°C; the surnatant was then adequately diluted with chromatographic mobile phase (15 mM of K_2_HPO_4_, pH 3.0). Neopterin and Creatinine levels were measured using a Varian pump (240, auto sampler ProStar 410) coupled to a fluorometric detector (JASCO FP-1520, λ_ex_ = 355 nm and at λ_em_ = 450 nm) for Neopterin and to a UV-VIS detector (Shimadzu SPD 10-AV, λ = 240 nm) for Creatinine determinations. Neopterin and Creatinine separations were performed at 50°C on a 5 μm Discovery C18 analytical column (250 x 4.6 mm I.D., Supelco, Sigma-Aldrich) at a flow rate of 0.9 mL^.^ min^-1^. The calibration curves were found to be linear over a concentration of 0.125^−1^ mmol^.^ L^-1^ and 1.25^−10^ mmol^.^ L^-1^ for Neopterin and Creatinine (the score in μmol^.^L^-1^ was divided by 88.4 to get mg^.^ dL^-1^ Creatinine) respectively. Inter-assay and intra-assay coefficients of variation were less than the 5%.

Interleukin 6-Urine (IL-6-U). IL-6-U levels were determined by Quantikine HS Human IL-6 Immunoassay (R&D Systems, Minneapolis, MN, USA), according to the manufacturer’s instruction. The method was previously described for plasma samples and the signal spectrophotometrically measured at a slightly different wavelength than in plasma (490 nm).

Nitrate/Nitrite levels. The determination of nitrate/nitrite levels in urine was performed by the spectrophotometric method to Griess reagent, utilizing a commercial colorimetric assay kit (Cayman Chemical, USA), at 545 nm by a microplate reader spectrophotometer (Infinite M200, Tecan, Austria). A linear calibration curve was obtained from pure nitrite and nitrate standards.

Urine Test Strip. The Urine Test Strips (Combi screen 11sys PLUS, GIMA, Gessate, Milan, Italy) was used to semi-quantitative determinations of ketones, pH, blood, urobilinogen, bilirubin, proteins, specific gravity/density and leukocytes in urine. Test was immediately performed in triplicate for each subject.

### Statistical Analysis

Statistical analysis was performed using the GraphPad Prism package (GraphPad Prism 6, Software Inc. San Diego, CA). Data are presented as means ± SD. Data were analysed using repeated Shapiro-Wilks W test. Experimental data were compared using ANOVA repeated measures with a Bonferroni post-hoc test. Paired t-test was used to test for differences between Pre and Middle-Race in the group of athletes who dropped out of the race. Pearson’s product moment correlation coefficient (r) with 90% confidence intervals (CI) was used to examine the relationships between selected parameters. Prospective calculation of power to determine significant number was made by using the Freeware G*Power software (http://www.psycho.uni-duesseldorf.de/abteilungen/aap/gpower3/). At a power of 80%, the calculated number of significant subjects was 11, well below the subject’s population recruited for this study.

## Results

### Anthropometric, physiological and haematological parameters

Twenty-five athletes (age 45.5±9.4 yr, height 1.76±0.05 m) finished the MUM in a mean time of 111:38±14:36 hours. Twenty-one (age 44.4±8.1 yr, height 1.76±0.05 m) dropped out of the race. Only eight of these latters arrived in Donnas (Middle-Race, 148.7 km). Therefore, thirteen runners dropped out the race before reaching the Middle Race point in Donnas. On these subjects it was impossible to collect the data. Therefore the parameters at Middle-Race were obtained from the subjects (n = 8), who reached the intermediate point in Donnas. All parameters investigated in the present study at the Pre-, Middle and Post-Race were collected at the same time. Herein after and throughout the text, the group of the runners who finished and the group of runners who dropped out of the race will be referred to as FR and NFR, respectively. The anthropometric parameters of the athletes, both altogether and in the two groups, are reported in Table 1. No significant differences between FR and NFR at Pre-Race were found, while a significant decrease of both weight and body mass between Pre-Race and both Middle and Post-Race was observed in the FR group. All significance levels are reported in the Table.

Physiological parameters of all athletes are reported in Table 1 as well. No significant differences between FR and NFR groups were found at Pre-Race. By contrast, in FR, with respect to Pre-Race, Heart Rate significantly increased at both Middle and Post-Race (+18% and +19% respectively), while systolic and diastolic blood pressures were found increased at Post-Race (+12%, +13%, respectively). In the NFR group a significant increase in Heart Rate (+29%) and diastolic blood pressure (+11%) was found at Middle- with respect to Pre-Race.

Haematological parameters of all subjects are reported in Table 2. No significant differences in glucose concentration and haematocrit values were found between the two groups at Pre-Race. By contrast, with respect to Pre-Race, haematocrit significantly decreased in both groups (FR (P<0.0001) and NFR (P<0.001)) at Middle-Race, while, in the FR group, the parameter was found decreased at Post-Race (-11.2%) too.

**Table 2:** Haematological parameters.

	ALL ATHLETS (n = 46)	FR (n = 25)			NFR (n = 21)	
	Pre-Race	Pre-Race	Middle-Race	Post-Race	Pre-Race	Middle-Race(n = 8)
**Lactate [La]**_**b**_ **(mM)**	1.91±1.08	1.57±0.52	2.97±1.04§	4.55±1.44¶	2.55±1.55	5.34±1.13§
**Glucose (mmol** ^.^ **L** ^**-1**^ **)**	113.45±14.6	111.72±9.97	117.12±11.36	115.92±14.77	114.51±13.2	116.14±18.47
**Hct (%)**	45.17±2.86	45.2±2.7	41.58±2.54 *	40.16±2.68	45.10±3.37	42.80±3.15§

Mean values (±SD) of the parameters collected from the athletes: altogether at Pre-Run, or referred to the FR and NFR groups at different racing times. [La]_b_ Blood lactate concentration; Hct: Hematocrit. Significant differences compared to Pre-Race: P<0.05 (* symbol), P<0.001 (§ symbol), P<0.0001 (¶ symbol).

Lactate concentration values resulted significantly different in the two groups (P<0.01) when referred to Pre-Race: +89% in FR and +109% in NFR at Middle-Race; +190% in FR at Post-Race.

### ROS production, oxidative damage and antioxidant capacity

**Fig 2:**
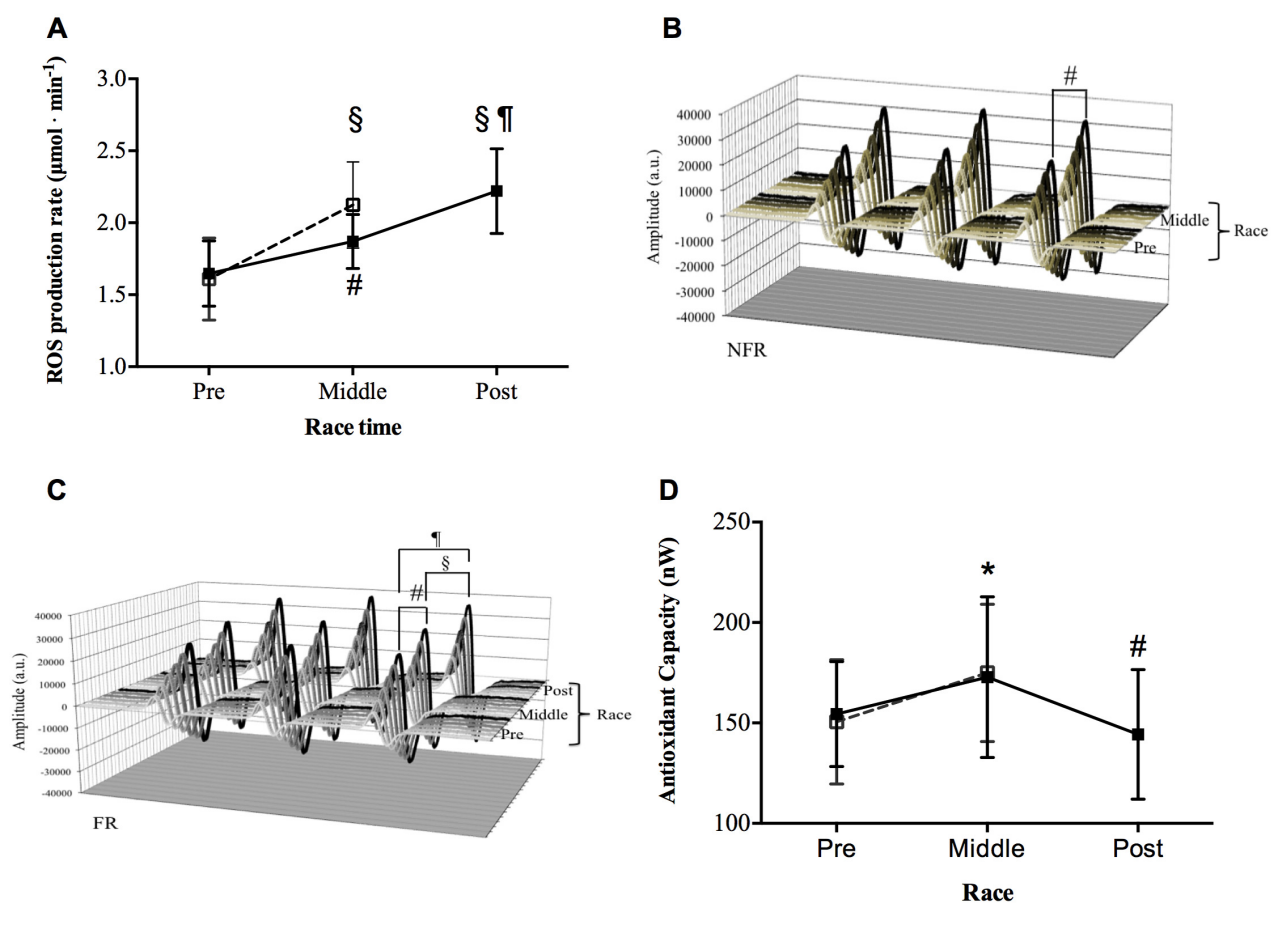
Effects of MUM on ROS Production and Antioxidant Capacity. The data obtained in the two groups of athletes are shown: FR (closed symbols), NFR (open symbols). Results are expressed as mean ± SD. Time course of: A) ROS production rate (μmol.min^-1^) detected by EPR technique before (Pre), at Middle and immediately Post race. Changes over time were significant compared to pre competition in both groups (# symbol, P<0.01 and § symbol, P<0.001); in the FR group: Pre with respect to Post-Run P<0.0001 (¶ symbol); Middle with respect to Post-Race P<0.001 (§ symbol). Examples of the stacked plots of the EPR spectra recorded: B) at Pre, and Middle Race collected from a NFR subject and C) at Pre, Middle and Post-Race in a FR subject. In each panel the increase of the signal amplitude (a. u.) is shown. The differences between Pre vs Midlle-Race (# symbol, P<0.01), Middle versus Post-Race (§ symbol, P<0.001) and Pre versus Post-Race (¶ symbol, P<0.0001) are indicated. D) Time course of the antioxidant capacity (nW) before (Pre), at Middle and immediately Post-Race. In the FR group, the changes over time were significantly different (* symbol, P<0.05) at Middle with respect to Post-Race and (# symbol; P<0.01;) with respect to Pre-Race. In Panels A and D, the lines are drawn to guide the eye.

The MUM induced a wide increase of ROS production in capillary blood. The time course of the ROS production rate levels (mean ± SD) in the two groups of runners is shown in Fig 2A. Starting from not significantly different basal levels, the ROS production significantly increased at Middle-Race (P<0.01) in both the FR (1.87±0.18 vs 1.65±0.22 μmol^.^min^-1^) and the NFR (2.13±0.29 vs 1.62±0.28 μmol^.^min^-1^) groups. Moreover, in the FR, ROS level was found significantly increased at the end of the race with respect to both Pre-Race (P<0.0001, Post-Race level: 2.20±0.27 μmol^.^min^-1^) and Middle-Race (P<0.001). The significant increase of the signal amplitude (a. u.) can be observed in the stacked plots, shown as an example, of the EPR spectra recorded at Pre, and Middle Run in a NFR subject (Fig 2B) and at Pre, Middle and Post-Race in a FR subject (Fig 2C).

Fig 2D shows the Antioxidant Capacity levels at the three race times. As can be observed, in the FR group, with respect to the level measured at Pre-Race (154.50±26.12 nW), this parameter results significantly increased at Middle-Race (172.80±39.98 nW, P<0.05), while decreased at Post-Race (144.30±32.26 nW, P<0.001). At Middle-Race, the parameters were found significantly increased (P<0.05) in the NFR group too (Pre- vs Middle-Race levels: 147.40±30.97 vs 179.00±30.17 nW).

**Fig 3:**
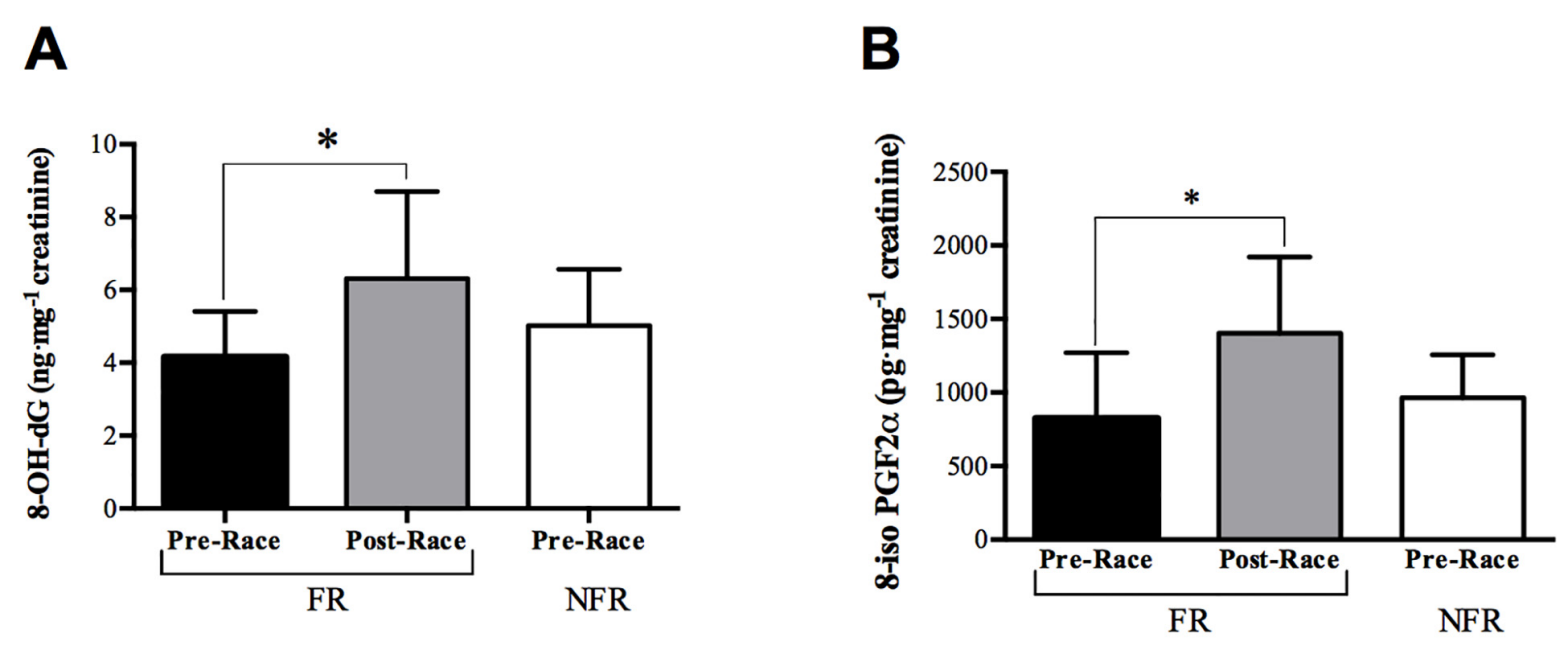
Effects of MUM on Oxidative damage biomarkers. Histograms of: A) 8-hydroxy-2-deoxy Guanosine (8-OH-dG, ng^.^mg^-1^ creatinine); B) 8-isoprostane (8-iso PGF2α, pg^.^mg^-1^ creatinine) concentrations in the FR and NFR groups at Pre and Post-Race. Results are expressed as mean ± SD;* significant differences (P<0.05) compared to Pre-Race.

The trend of the oxidative damage biomarkers is displayed in the histograms of Fig 3A (8-hydroxy-2-deoxy Guanosine) and Fig 3B (8-iso PGF2α) at Pre and Post-Race times. No significant differences were found between the levels calculated for the FR and NRF groups at Pre-Race. As can be observed in the figure, at Post-Race in FR, both markers attain a significantly greater level (P<0.05): 8-OH-dG/creatinine (Post- vs Pre-Race levels 6.32±2.38 vs 4.16±1.25 ng^.^mg^-1^) and for 8-iso PGF2α/creatinine (Post- vs Pre-Race levels 1404.0±518.30 vs 822.51±448.91 pg^.^mg^-1^).

### Inflammatory state

**Fig 4:**
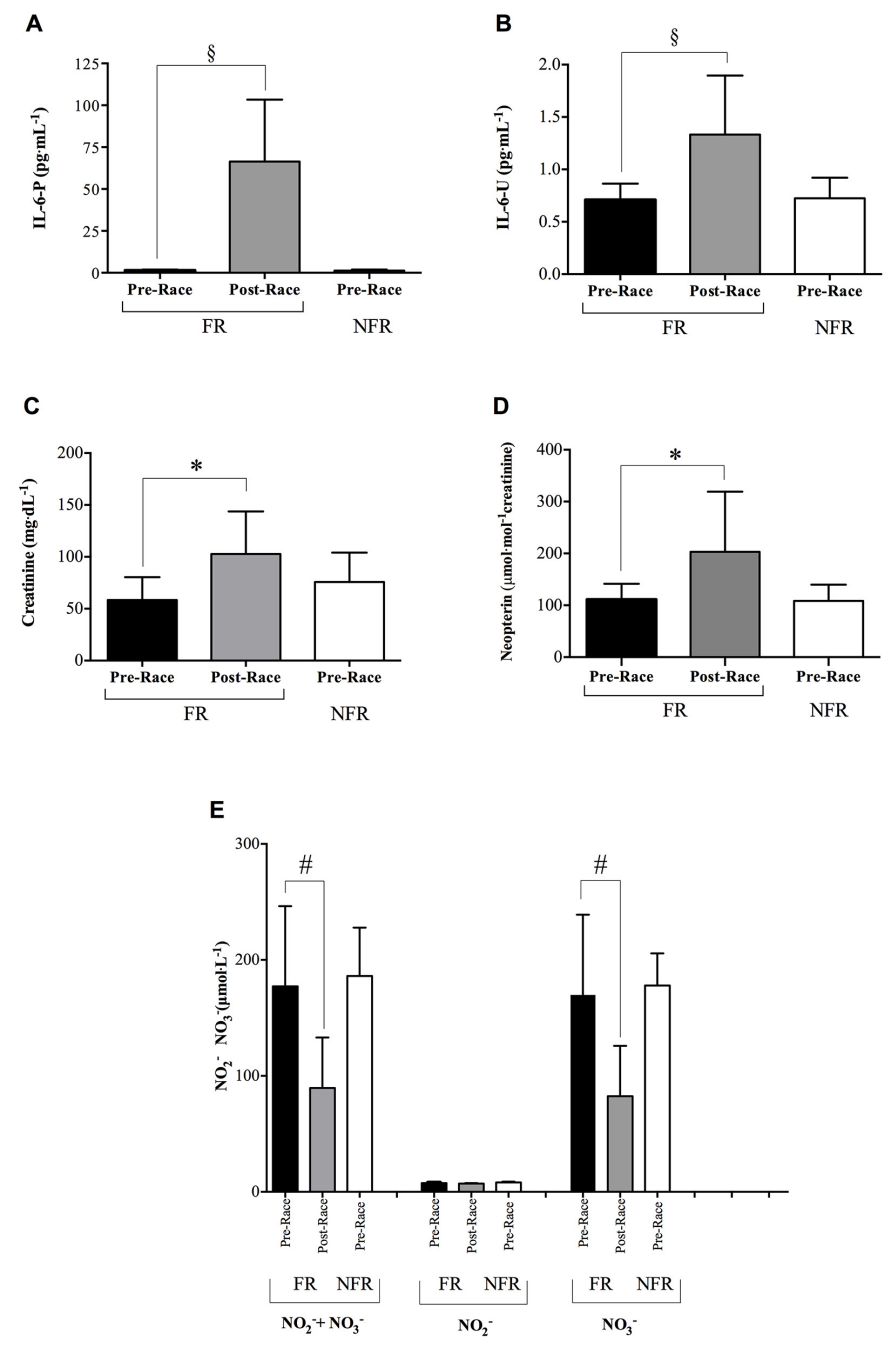
Effect of MUM on the inflammatory state. Histograms of: A) IL-6-P (pg^.^mL^-1^), B) IL-6-U (pg^.^mL^-1^), C) Creatinine (mg^.^dL^-1^), D) Neopterin (μmol^.^mol^-1^ creatinine) levels, and E) NO metabolites: Nitrite (NO_2_^-^) and Nitrate (NO_3_) concentrations, in the FR (full bars) and NFR (empty bars) groups. Results are expressed as mean ± SD. Statistically significant differences symbols: P<0.05 (*); P<0.01 (#), P<0.001 (§) compared to Pre-Race.

According to Fig 4, a significant increase (P<0.001) in the inflammatory state was suggested in the FR group by the IL-6-P (Fig 4A) and IL-6-U (Fig 4B) levels calculated at Post-Race (66.42±36.92 and 1.33 ± 0.56 pg^.^mL^-1^ respectively), with respect to Pre-Race (1.29±0.54 and 0.71±0.17 pg^.^mL^-1^ respectively).

#### Creatinine and Neopterin

The histogram panels of Fig 4C and 4D (Creatinine and Neopterin/creatinine levels respectively) suggest a urinary dysfunction at Post-Race with respect to Pre-Race in FR (full bars). In fact, the levels of both the biomarkers of renal function significantly increased (P<0.05): Creatinine: 102.80±41.01 vs 58.47±21.91vs mg^.^dL^-1^ (Post- vs Pre-Race); Neopterin: 203.20±115.81 vs 111.20±30.41 μmol^.^mol^-1^creatinine (Post- vs Pre-Race). By contrast no difference was found in the basal levels measured in the two groups (empty bars).

#### Nitrite and Nitrate concentration

Fig 4E shows the urinary NO metabolite ((Nitrites (NO_2_^-^), Nitrates (NO_3_^-^)) levels (μmol^.^L^-1^) and their sum measured in the FR group. Starting from a total level (NO_2_^-^ + NO_3_^-^) of 177.2±69.24 μmol^.^L^-1^, the concentration can be observed to decrease significantly (P<0.01) at Post-Race 89.58±43.49 μmol^.^L^-1^. As well known, this finding has to be ascribed to the significant decrease (P<0.01) of NO_3_^-^ alone: Pre vs Post-Race: 169.70±79.39 vs 82.46±43.47 μmol^.^L^-1^. By contrast, as can also be observed in the figure, the NO_2_^-^ concentration did not change significantly: 7.49±0.55 μmol^.^L^-1^ at Pre vs 7.12±0.45 μmol^.^L^-1^ at Post-Race time. A high level of urinary NO metabolites (NO_2_^-^ + NO_3_^-^185.3±73.16 μmol^.^L^-1^) at Pre-Race was found in the NFR group too (see Fig 4E).

#### Urine Test Strips

Urine standard parameters are reported in **Table 3.** A significant increase at Post vs Pre-Race in FR group was found as concerning: bilirubin (33.36±22.59 vs 18.22±9.32 μmol^.^L^-1^), urobilinogen (33.16±16.50 vs 24.06±9.28 μmol^.^L^-1^), ketones (2.00±1.87 vs 0.5±0.01 mmol^.^L^-1^), proteins (87.27±28.32 vs 24.09±16.44 mg^.^dL^-1^), erythrocytes (12.70±16.77 vs 4.10±2.38 Ery^.^ μL^-1^), leucocytes (36.60±25.03 vs 18.60±11.86 Leuko^.^ μL^-1^) and specific gravity (1.03±0.01 vs 1.02±0.01) levels. By contrast, in the two groups, the basal levels did not result significantly different.

**Table 3:** Urine standard analysis.

	ALL ATHLETES (n = 46)	FR (n = 25)		NFR (n = 21)
	Pre-Race	Pre-Race	Post-Race	Pre-Race
**Bilirubin (μmol** ^.^ **L** ^**-1**^ **)**	18.29±9.07	18.22±9.32	33.36±22.59§	18.58±8.73
**Urobilinogen (μmol** ^.^ **L** ^**-1**^ **)**	24.48±9.22	24.06±9.28	33.16±16.50#	26.25±9.58
**Ketones (mmol** ^.^ **L** ^**-1**^ **)**	0.56±0.35	0.5±0.01	2.00±1.87§	0.83±0.81
**Proteins (mg** ^.^ **dL** ^**-1**^ **)**	24.09±16.44	24.09±26.44	87.27±28.32¶	12.50±11.29
**Erytrocytes (Ery** ^.^ μ**L** ^**-1**^ **)**	4.43±2.47	4.10±2.38	12.70±16.77*	5.83±2.58
**pH**	5.32±0.52	5.5±0.59	5.364±0.50	5.00±0.03
**Leucocytes (Leuko** ^.^ μ**L** ^**-1**^ **)**	23.06±17.59	18.60±11.86	36.60±25.03#	41.67±25.82
**Specific gravity**	1.02±0.00	1.02±0.00	1.03±0.00*	1.03±0.00

Mean (±SD) values of the variables measured by a urine test strip from the athletes: taken altogether and NFR groups at Pre-Race and FR at Pre and Post-Race.

Statistically significant differences symbols: P<0.05 (*), P<0.01 (#), P<0.001 (§l) and P<0.0001 (¶).

**Fig 5:**
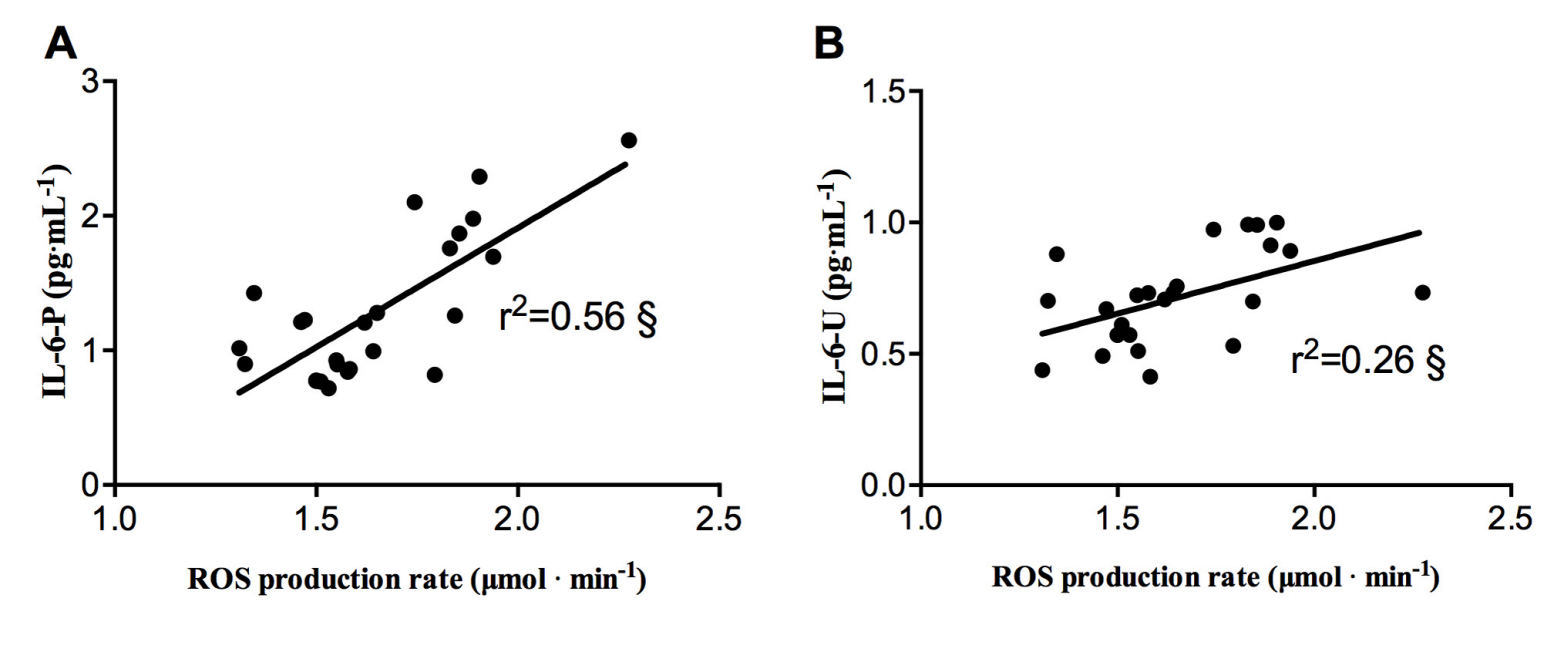
ROS-Interleukin correlation at Pre-Race in FR. Plots of ROS production rate levels (μmol^.^ min^-1^) versus: A) IL-6 in plasma (IL-6-P) and B) IL-6 in Urine (IL-6-U). The significant (§ P<0.001) linear relationship (solid line) is also shown and the correlation coefficient (r^2^) reported in each panel.

Finally, the correlation between ROS production and plasma and urine Interleukin-6 is displayed in Fig 5. In the FR group at Pre-Race, by Pearson’s product-moment, a linear correlation between: ROS production rate and A) IL-6-P (r^2^ = 0.56, P<0.001) and IL-6-U (r^2^ = 0.26, P>0.05, panel B) was found.

At Post-Race, a correlation between: ROS production rate and i) antioxidant capacity (r^2^ = 0.33, P<0.05); ii) lactate (r^2^ = 0.14, P>0.05); iii) IL-6-P (r^2^ = 0.17, P>0.05) was estimated by Pearson’s product-moment as well (Data not shown).

## Discussion

This study adopted micro-invasive methods (just capillary blood by fingertip and urine were collected for the measurements) aiming to gain a detailed insight into the physiological mechanisms involved under extreme conditions, when the human body is pushed to its limits. This choice becomes of paramount importance when studies are carried out on athletes and even more under extreme environments [31]. A group of experienced ultra-marathon runners was monitored throughout the evaluation of several OxS markers: ROS production, oxidative damage, inflammatory response and functional renal impairment, coupled with anaerobic response analysis.

As widely reported, during intense exercise, the significant need for ATP production is associated with a large oxygen flux into mitochondria of the working skeletal muscles. It’s well known that intense physical exercise enhances oxygen consumption together with ROS production [32,33]. Oxidative stress, inflammation and temporary impairment of the renal function are associated with fatigue and recovery from endurance exercise. Finally OxS, which represents an imbalance between oxidants, coming from ROS production, and antioxidants in favour of the former ones, leads to a disruption of redox signalling and controlling proteins and DNA with consequent oxidation of lipids [34].

In the present study, despite the similar baseline ROS production rate values found in both FR and NFR groups, a significant and different increase of this parameter was detected in the two groups (FR (+13%); NFR (+32%)) at Middle with respect to Pre-Race. Moreover it's important to note that in NFR at Middle-Race the ROS production concentration rate was found even higher than the level measured in the FR group at Post-Race (+33%, see Fig 2A, 2B and 2C). The overproduction of ROS observed in the NFR group may be due, among others, to the heavy contribution of the anaerobic metabolism in the race economy of these subjects, as demonstrated by the correspondent significantly higher lactate concentration found in this group (see Table 2). Indeed, ROS can result from the increased xanthine oxidase activity triggered by transient hypoxic conditions that may occur even during predominantly aerobic exercise due to blood-redistribution. Xanthine oxidase enzyme is present in most human tissues, including skeletal muscle, and/or cells of the immune system such as neutrophils, monocytes, macrophages that are all activated in response to exercise [35]. Moreover, the positive significant relationship (r^2^ = 0.14) between lactate concentration and ROS production at Post-race seems to confirm the hypothesis of Nikolaidis et al. [36] that muscle damage during exercise would also be triggered by OxS. Nonetheless, in the present study, muscle damage as a consequence of the ultra-endurance exercise is also suggested by the significant increase (+76%) of the urinary Creatinine level at Post-race (see Fig 4C). Altogether, the results show that mountain ultra-endurance exercise causes an oxidative insult. Indeed the significant increase in both FR and NFR groups of the OxS parameters further confirms this finding. Likewise other authors [37] also reported a significant increase of the oxidative stress in response to marathon and ultra-marathon races.

In accord to Turner et al. [4], the significant increase of the DNA damage (see Fig 3A **)** at the end of the race (+52%) is attributable to the increase of the ROS production rate. Also the significant increase of 8-isoprostanes (+71%) can be considered a response to the strenuous exercise requested by this mountain ultra-marathon race (see Fig 3B **)**. Isoprostanes are produced in vivo by the free radical-catalyzed oxidation of polyunsaturated fatty acids bound to membrane [38]. In addition the increased lipid oxidation observed at Post-Race may be related to the increased availability of plasma-free fatty acids, which are needed to support the long competition metabolic requests, as also reported in the literature [39]. As a matter of facts, the whole body fat mass was found to significantly decrease progressively during MUM (see Table 1). Moreover, as also reported in the literature, intense exercise increases lipid peroxidation products, whereas provision of antioxidant compounds decreases them [40]. Thus, a good antioxidant capacity might allow the system to respond adequately to the intense physical activity required by the race. The increase of the antioxidant capacity observed at Middle-Race can be viewed as an index of the activation of the body’s antioxidant mechanisms in plasma, when considering the mobilization of the tissue antioxidant stores into plasma as one possible responsible mechanism [41]. Indeed this is a widely accepted phenomenon that would help to maintain or even increase plasma antioxidant status upon an increased production of ROS [42]. In our study, the antioxidant capacity was not considerably affected by the stress produced by the race. Indeed, only at the end of the race (Post-Run), after an average of 111 hours of running, the system began to loose (-7% in antioxidant capacity) its ability to respond to the increased production of ROS (see Fig 2D). This finding suggests a good compensation of the increased oxidative stress by the antioxidant defense system in these runners [5].

Exercise-induced oxidative stress is also an important mechanism causing injury to erythrocytes during and after strenuous physical exercise, as confirmed by the evidence of an increased RBC turnover in non-traumatic sports such as swimming, cycling, rowing, and weight-lifting [43]. Actually, our results showed a significant decrease during the race (Middle-time) of the mean hematocrit value in both the groups of athletes. Hct decreased in the FR at Middle- (-8%) and Post-Race (-11.2%) and in the NRF group at Middle-Race (-5%) (see Table 2), in well agreement with the results reported by Klapcinska et al [44]. Erythrocytes disruption was mirrored in the significant increase of urinary bilirubin and urobilinogen concentrations here reported (see Table 3), suggesting an heme degradation secondary to hemolysis. As a matter of facts, plasma volume variation is a really common effect in endurance sports [45], and this possibility would be taken into account when analyzing and interpreting hematocrit variation data. Maximal and submaximal exercises, either of short or long duration, almost always increase blood viscosity due to a rise of plasma viscosity itself and/or hematocrit. Maximal exhaustive exercise induced significant increments of RBC, Hb, Hct, and white blood cells (WBC) together with a decrement of plasma volume. These findings could be ascribed to: local plasma water-shift mechanisms and especially to a transient shift of plasma water from intra- to extravascular compartments, redistribution of red cells (RBC) in the vascular bed, enrichment of plasma in several proteins, coming presumably from lymphatics, loss of water through thermoregulatory sweating, entrapment of water into muscle cells. In Finishers (FR), total body water (TBW, see Table 1) did not change during or immediately after the race, so that the reduction of Hct may be attributable to a reduction of the blood corpuscular components (i.e. principally RBC). This finding together with the presence of erythrocytes in urine and the increase of the urinary bilirubin level suggested us the correlation between hematocrit variation and erythrocytes disruption or hemolysis. Indeed the degradation of myoglobin occurring during the race could also contribute to bilirubin production [46]. This latter evidence may further support the hypothesis of muscle degradation. As mentioned above, intense and strenuous exercise increases OxS and this process stimulates cytokines production from various cell types and un-regulates the inflammatory cascade. It has been consistently shown that circulating levels of interleukins (IL)-6,-8,-1, receptor antagonist (IL-1ra) and IL-10 increase remarkably following endurance exercise longer than 2h such as marathon [22]. IL-6 is the first cytokine present in the circulation during exercise-contracting skeletal muscle that is the main source of plasma IL-6. Its level was found to increase up to 100-fold, depending on intensity and duration of the endurance exercise [22]. According to the literature [22,47], the results at the end of the race in FR demonstrated an inflammatory response, stimulating the synthesis of IL-6-P (+5031%) as well as the excretion of IL-6-U (+83%) (see Fig 4A and 4B), compared with Pre-Race values. Literature data suggest that plasma IL-6 response is related to the muscle glycogen content. This observation suggests that the production and release of IL-6 from contracting skeletal muscle is related to glycogen depletion occurring during prolonged exercise [48]. The present study investigated and significantly (p<0.001) found at Pre-Race the relationships between ROS production and IL-6-P (r^2^ = 0.56) and Il-6-U (r^2^ = 0.26) (see Fig 5A and 5B). As previously reported [31], our findings also support such correlation between variables of oxidative stress and cytokines. After MUM the measured IL-6-P values correlate with the ROS production rate (r^2^ = 0.16): this finding proposes the inflammatory cascade as a related contributor of ROS production during prolonged exercise.

An increase in systemic inflammation, besides the markers of oxidative stress, can also be associated to altered biochemical parameters measured in urine and in particular to Neopterin concentration level, that has previously been shown to indicate an enhancement of immune response [49]. Recently, high Neopterin production has been associated with immune cellular activation and increased production of OxS [50]. Therefore, throughout Neopterin assessment, not only the extent of cellular immune activation, but also the extent of OxS can be estimated [23]. Increase of urinary Neopterin concentration was observed in the present study immediately after race (Post-Race, +83%), associated with rising of ROS production and lowering of antioxidant concentration (see Fig 5B). Therefore Neopterin can be also regarded as a marker of the ROS formed by the activated cellular immune system. Findings of temporary "impairment of renal function" could also be considered as a likely physiological response to mountain ultra-marathon race. Exercise-induced proteinuria is generally benign and related to the intensity rather than to the duration of the exercise [51]. As expected, Post-Race total proteinuria significantly increased (+262%) as well as bilirubin and urobilinogen levels. Red and White blood cells were found dramatically increased too (see Table 3). As a final remark, physical exercise is well known to increase blood flow and shear-stress, by enhancing the vascular function, throughout the production of nitric oxide. Inorganic nitrate and nitrite have benefits in terms of cardiovascular regulation and modulation of mitochondrial function, which plays a central role in physical performance and health [52]. Therefore the determination of urinary nitric oxide metabolites, reflecting the levels of nitric oxide in the blood [53] is of interest, in particular, as biomarkers of the endogenous NO synthesis from L-Arg at kidney and/or whole body level. Basal data of the urinary excretion rate of nitrate and nitrite recorded at Pre-Run were found even higher than those recorded in healthy humans following an uncontrolled nitrate diet [54]. Indeed regular long-term training is able to provoke training-induced up-regulation of nitric oxide metabolite levels [55]. Nitrates significantly decreased upon intense and strenuous exercise as reported by others too [56]. Because nitrate elimination mirrors endogenous NO production, lower levels of urinary NO metabolites may be related to lower levels of physical performance. Indeed a decrease in green leafy vegetables intake, the reduction of the number of erythrocytes, the major intravascular storage site of nitrite in human blood [57], and the endogenous NO synthesis from L-Arg may at the same time be possible explanations of the observed urinary nitric oxide metabolites reduction.

## Limitations of the Study

The authors are aware that the current study suffers from certain limitations. Among them the most relevant is the need of a more detailed analysis of the markers of oxidative stress and antioxidants. Nonetheless, novelty of the current work, was the use of micro-invasive analytic techniques. Aiming at minimizing the invasiveness of the method and hence to improve its potential use for routine applications, oxidative stress markers determination, requiring more invasive venous blood samples, was herein avoided. This choice was also supported by the linear correlation between ROS production rate and the principal biomarkers’ concentration previously observed [17,18]. It goes without saying that the choice of using capillary blood limited the amount available to perform measurements and then the number of parameters suitable to be analyzed. Moreover there was no chance of calling back the participants, coming from all over Italy, in order to extend the study to the post race recovery. Therefore mechanisms finding how oxidative stress is implicated in the recovery phase were not included in this study.

On the other hand, as already pointed out, no limitations were imposed to the participants concerning the use of food, vitamin/minerals supplements, herbs and drugs. Thus nutritional data about food intake before and during the race could not be reported in this study. At the same time, as is well known, the nutritional intake interferes with the antioxidant capacity of the subject. Nonetheless, the questionnaire administered at pre-race attested that none of the runners assumed antioxidants, and even if no specific dietary protocols were imposed to the participants, the suggested scheme about proteins and carbohydrates assumption was generally followed by the athletes. Finally, during the race, food and water were provided by the organizers. All together, these observations lead us to conclude that, even if an effect on the measured data of the diet and the attitude towards drugs could not, on principle, be excluded, it did not play a pivotal role. Finally it is worth noting the influence of the weather conditions on the performance of the runners, even if their extent could not, of course, be quantified.

## Conclusions

In the present study, we confirmed that intense endurance, as mountain ultra-marathon race, caused a damage in the oxidative stress balance, highlighted by the assessed ROS overproduction, changes in plasma and urine cytokines (IL-6) levels, hematologic and standard urine parameters. One of the most important finding is that the antioxidant capacity was not considerably affected by this kind of exercise, although it appeared to be weaker among the athletes from the NFR group: at mid-race, the non-finishers were characterized by lower antioxidant capacity and a higher levels of oxidative damage biomarkers, compared with the finishers. The results suggested important observations of significant increases in 8-OH-dG and 8-isoPGF2α, attributable to exercise- induced oxidative DNA and membrane polyunsaturated fatty acid (PUFA) damage, associated with marked enhancement of the immune response, as reflected by several-fold increase in plasma IL-6 and almost two-fold rise in urine IL-6 and urine Neopterin/Creatinine ratio at the finish of the race. A higher value of lactate was observed in the NRF group at Middle-Race too.

In synthesis, these results lead us to conclude that the differences between the two groups were ascribable to a less efficient metabolic economy in the NRFs, turning to anaerobic sources and higher ROS production.

The relationships among the variables determined in the plasma (i.e ROS and IL-6-P) and urine (IL-6-U) at Pre-Race were of physiological relevance. Nonetheless further investigation is required to highlight the mechanisms of the relationship between ROS production and/or cytokine, hematological value following prolonged endurance exercise.

Finally it is worth noting the reliability of the micro-invasive methods adopted in the present study, mostly suitable to be carried out in paramount situations like the ultra-marathon race environments and at the same time leading more readily the athletes to undergo scientific tests.
